# The Molecular Biology of Soft-Tissue Sarcomas and Current Trends in Therapy

**DOI:** 10.1155/2012/849456

**Published:** 2012-05-10

**Authors:** Jorge Quesada, Robert Amato

**Affiliations:** ^1^Division of Oncology, Department of Internal Medicine, University of Texas Health Science Center at Houston (Medical School), Houston, TX 77030, USA; ^2^Memorial Hermann Cancer Center, The University of Texas, 6410 Fannin Street, Suite 830, Houston, TX 77030, USA

## Abstract

Basic research in sarcoma models has been fundamental in the discovery of scientific milestones leading to a better understanding of the molecular biology of cancer. Yet, clinical research in sarcoma has lagged behind other cancers because of the multiple clinical and pathological entities that characterize sarcomas and their rarity. Sarcomas encompass a very heterogeneous group of tumors with diverse pathological and clinical overlapping characteristics. Molecular testing has been fundamental in the identification and better definition of more specific entities among this vast array of malignancies. A group of sarcomas are distinguished by specific molecular aberrations such as somatic mutations, intergene deletions, gene amplifications, reciprocal translocations, and complex karyotypes. These and other discoveries have led to a better understanding of the growth signals and the molecular pathways involved in the development of these tumors. These findings are leading to treatment strategies currently under intense investigation. Disruption of the growth signals is being targeted with antagonistic antibodies, tyrosine kinase inhibitors, and inhibitors of several downstream molecules in diverse molecular pathways. Preliminary clinical trials, supported by solid basic research and strong preclinical evidence, promises a new era in the clinical management of these broad spectrum of malignant tumors.

## 1. Introduction

Remarkable gains in the understanding of cancer biology have been attained in the past two decades. Novel methodologies and laboratory techniques have allowed molecular dissection of cancer cells leading to a more precise portrait of tumorigenesis. The biological and molecular characteristics of transformed cells that produce and sustain malignant growth have been organized in a coherent and comprehensive manner by Hanahan and Weinberg [1]. Sustained growth, evasion of growth suppressors, resistance to death, induction of angiogenesis, and the ability to invade and spread are fundamental tumor characteristics, all of which have underlying molecular correlates that scientists are beginning to unravel and understand. More recently, other enabling characteristics have supplemented these initial concepts, namely, avoidance of immune destruction, tumor-promoting inflammation, deregulation of cellular energy pathways, and genomic instability [1].

 Although these principles are applicable to all malignancies, individual classes of tumors and even individual patients differ in the particular specificities of the complex process of malignant growth. Further, this is a multistep dynamic process subject to change and adaptation over the course of the disease, from premalignant lesions to metastatic spread.

Sarcoma research has lagged behind other cancers because of the rarity of these tumors and the multiple clinical and pathological entities that compose these malignancies. Yet, research work with sarcomas has been central in elucidating many of the modern concepts of cancer biology including the molecular signals driving tumor growth and permanence. Even though few advances in the treatment of these highly resistant tumors have occurred, some milestones have been carved based on clinical and bench research in sarcoma. This paper highlights some of the most outstanding work in the past, current knowledge of the molecular biology of sarcomas and the challenges to control or cure these rare, heterogeneous malignancies.

### 1.1. The Rous Sarcoma Virus

The seminal paper by Rous describing the ability to transfer an avian spindle cell sarcoma from one Plymouth Rock hen to another was largely dismissed as irrelevant to human disease [2]. Rous described experiments that eventually led to our modern understanding of the genesis of cancer. Later, Rous published work describing the transmission of sarcoma using tumor cell-free extracts indicating that a biological agent on the filtrate could cause tumor growth and could be propagated through subsequent passages. These observations opened the field of tumor virology and would link sarcoma research intimately to the field of cancer research.

The agent responsible for this unprecedented discovery was a retrovirus (Rous sarcoma virus (RSV)). A cadre of researchers began to unravel the mystery of this finding over the years. Remarkably, it took more than 50 years from the initial report for the world to realize the magnitude of the discovery when the existence of a genetic sequence in the RVS capable of inducing transformation, the src gene was discovered. The src-encoded tyrosine kinase (TK) was the first evidence of TK activity involved in malignant transformation [3], and it was the first to demonstrate that activation occurs by phosphorylation of the aminoacid tyrosine in host cell proteins. These enzymes have been shown to be essential for the malignant transformation of cells by oncogenic signals [4]. Steadily the functional relationship between oncogenic protein activity and receptor signaling began to emerge into a cohesive model that occupies much of the current research in carcinogenesis and biology of cancer cells. The eventual development of targeted therapies, interfering with these molecular pathways saw the dawn of a modern approach to cancer therapy.

The viral etiology of cancer in vertebrates evolved from these observations [5]. Viral particles were described in multiple vertebrate species and culminated with the identification of the first retroviral oncogene named “v-src” and its cellular homologue (c-src). It was realized that the viral oncogenes derived from functional cellular genes (protooncogenes) capable of inducing malignant transformation upon activation [6]. Subsequent years saw the identification of a number oncogenes and tumor-suppressor genes that were altered in human cancer. The concept of clonal evolution and a molecular model of multistep tumorigenesis evolved from these discoveries [7].

### 1.2. RAS and Human Oncogenes

As the concept that cancer was a disease of altered genes gained momentum, research with other animal sarcoma-inducing viruses led to further remarkable discoveries. The viruses that stand out were the rat sarcoma virus from the 1960s. The ras genes from the Kirsten sarcoma virus (KRAS), the Harvey sarcoma virus (HRAS), and later NRAS were shown to encode highly related proteins (H-Ras, N-Ras, K-Ras4A, and K-Ras4B) [8]. Later on, the cellular homologous of KRAS was found in human cancer cells (1982). A single aminoacid mutation was proven to allow the constitutive activation of the oncogene. Thus, the concept of cellular oncogenes capable of inducing human cancer was confirmed by the cloning of c-RAS the first human oncogene. The Ras proteins were later found to be GTPases involved in cellular signal transduction for cell growth and division. Activating mutations in Ras have been described in 20–25% of all human tumors and much higher in some specific tumor types [9]. During the 1990s the role of KRAS in tumorigenesis was established and the molecular signaling pathways involved in this process began to be unraveled [10].

Enormous scientific gains in the understanding of human cancer derived from the study of these viral agents. These discoveries, derived from the study of sarcomas, will remain engrained in the annals of history of cancer.

### 1.3. Coley's Vaccine

The first systematic study of immunotherapy for the treatment of malignant tumors began in 1891 by William Coley. Following the clinical observation of a dramatic response of one young patient to an injection of streptococcal organisms Coley treated, amidst much criticism and controversy, hundreds of patients with soft-tissue and bone sarcomas over a span of four decades [11]. Later he would use heat-killed streptococcal organism and *Bacillus prodigiosus* (*Serratia marcescens*), a concoction that became known as Coley's Toxin. By 1920 his work would become of ill repute. The Bone Sarcoma Registry (the first established cancer registry) declared lack of sufficient evidence of the therapeutic benefit claimed by Coley. Regardless of the longlasting debate surrounding his work, Coley's research would set the foundations on cancer immunotherapy. The scientific interest on this approach will rapidly grow as the biology of the immune system was progressively better understood. We could now assume that the effects of the Coley's toxins were mediated by activation of the immune system, with ensuing cellular activation and production of powerful cytokines (e.g., interleukins, interferons, tumor necrosis factor, etc.), some of which would become therapeutic tools in contemporary cancer immunotherapy.

### 1.4. Sarcomas and Interferon

The discovery of interferon was dismissed as a laboratory curiosity until Gresser showed that human leukocytes produced substantial amounts of these cytokines and later described an antiproliferative effect on cultured cells and animals [12]. Immunomodulating properties were also ascribed to interferon (IFN) and triggered enormous interest to investigate IFN as a potential anticancer agent, amidst the growing evidence of the existence of cancer-inducing transforming viruses. Strander pioneered the initial clinical use of IFN in human cancer. His group showed *in vitro* evidence of the activity of IFN in osteosarcoma (OS) cells and in human sarcoma tumors transplanted into mice which led to studies using IFN treatment in OS after surgery [13]. At a decade of followup, sarcoma-specific survival was 38% for the patients treated before 1985 and 63% for the group treated with higher total IFN dose afterwards. Because of the uncontrolled study design, there is no way to confirm a direct clinical benefit from IFN therapy in this pioneering study. Yet, continuous interest remains in exploring the potential benefit of IFN in OS, and a prospective randomized trial was launched by the European and American Osteosarcoma Study Group (EURAMOS 1) using pegylated IFN. We know now that IFN is an endogenous antiangiogenic regulator. IFN-*α* mediates cellular signaling via the JAK/STAT cascade with complex interaction with other signaling pathways including MAPK, PI3K, mTOR, and IFN-induced apoptosis via activation of Bak and Bax and the mitochondrial pathway. All of these molecular cascades are seemingly necessary in generating an IFN response [14, 15].

### 1.5. Kaposi's Sarcoma: A Model of Viral Carcinogenesis

Kaposi's sarcoma (KS) was originally described in 1872 and for decades remained as an uncommon low-grade, slow-growing tumor affecting predominantly patients of Jewish and Mediterranean origin (“Classsic” KS). In the 1950s, an endemic type of KS was identified in equatorial Africa regions with some patients exhibiting rapid and aggressive course and high mortality. Epidemiological data from this endemic form raised suspicion of a viral etiology in association with increased relevance of oncogenic viruses in other malignancies [16].

Renewed efforts to identify the cause of KS came as a result of the high incidence of KS in patients with AIDS. Epidemiological data led to the proposal that a sexually transmitted agent was responsible for KS [17]. Distinct DNA fragments of a herpes virus from KS were first isolated in 1994 [18]. The new virus was found to be a gamma herpes virus and was designated *HHV-8*. HHV-8 DNA is found in over 90% of specimens from classic KS, endemic KS, posttransplant KS, and epidemic KS.

Expression of multiple cellular-derived oncogenes has been identified by genetic dissection of the HHV-8 during its latent and lytic phases [19, 20]. KS lesions contain predominantly HHV-8 in latent phase. In these cells, HHV-8 expresses a limited number of “latent” genes (Table 1). The lytic phase of HHV-8 is more closely implicated in the development of KS and its ability to spread from lymphoid tissue to endothelial cells. The switch to the lytic phase results in the expression of a number of virally encoded cellular homologues (Table 1). The gene expression during the lytic phase is initiated by the HHV-8 replication transactivation activator gene (ORF 50; RTA), which is under regulatory control by LANA-1. RTA activates DNA and protein synthesis resulting in replication and assembly of new infective viral particles [19]. Lastly, the role of HIV virus in AIDS-related KS has been studied and suggests a synergistic effect with HHV-8 in the genesis of the tumor in these patients. The HIV Tat protein has been shown to compete with heparin sulfate proteoglycans, which bind *β*-fibroblast growth factor (*β*-FGF), increasing free levels of this angiogenic factor. KS has been described as an angiogenic neoplasm, and the genetic profile of the spindle cells, a major cellular component of KS lesions, closely resembles lymphatic endothelium [21]. These endothelial cells are responsive to angiogenic growth factors. Second, HIV Tat activates HHV-8 increasing expression of several early viral genes (Table 2).

IFN was shown to produce antitumor effects in AIDS patients with KS [22, 23] leading to the FDA approval of IFN for treatment of AIDS-associated KS in 1988. However, it was the control of HIV replication with highly antiviral antiretroviral therapies (HAART) combinations that resulted in a significant decline in the incidence of KS in AIDS patients. Whether this was the result of suppression of HIV-Tat (or other HIV genes) or the partially restoration of immunity, this epidemiological shift reinforces the participatory role of HIV in the tumorigenesis of KS. 

KS stands out as one *bona fide* example of viral carcinogenesis in man. Although many factors are required for the malignant transformation to occur, HHV-8 is the primary trigger of a cascade of molecular events that lead to the development of this unique type of sarcoma.

### 1.6. Gastrointestinal Stromal Tumors (GISTs)

The term “gastrointestinal stromal tumors” (GIST) refers to mesenchymal tumors of the gastrointestinal tract with features of neurogenic or myogenic differentiation but neither of neurogenic (S100-protein negative) or of smooth muscle origin (absence of myofilaments). Somatic mutations of c-KIT are involved in the genesis of these tumors. The transforming viral gene, Hardy-Zuckerman 4 feline sarcoma virus (v-KIT; HZ4-FeSV), was discovered in 1983 in feline sarcomas [24]. The c-KIT gene is the cellular homologue of a v-KIT that encodes a TK growth factor receptor (CD 117) and has been associated with growth and differentiation of immature cells [25]. The CD117 receptor is expressed by the interstitial cells of Cajal (ICC) and by neoplastic cells most notably by GIST [26]. From these observations, it was concluded that the ICC was most likely the cell of origin of GIST. The binding of the ligand Steel factor (SLF), or stem-cell factor to CD117 results in activation of KIT TK and its downstream substrates, which serve as effectors of signal transduction. Mutations of c-KIT resulted in gain of function of the KIT TK and constitutive activation of its molecular downstream pathways, predominantly the PI3K pathway [27, 28].

Approximately 75–85% of GIST patients express activating mutations of KIT of which exon 11 is the most common (57–70%), followed by exon 9 (5–18%) and rarely exon 13 and 17 domains (<2%). Only 5% of patients exhibit mutations in PDGFR and some patients exhibit neither KIT nor PDGFR (10–15%) [29]. Work with a c-KIT expressing human myeloid leukemia cell line proved that STI 571 (imatinib mesylate) was able to block c-KIT autophosphorylation [30]. Shortly after, a human GIST cell line expressing an active KIT mutation was completely inhibited by imatinib [31]. These findings translated into the first report on the clinical efficacy of imatinib in a single patient with GIST [32] which was followed by an auspicious phase II trial [33] and a fast track FDA approval. Thus, GIST became the first solid tumor, where therapeutic interference of a mutated TK mutation showed clear benefit.

Later work demonstrated that the specific location and nature of the activating mutation on KIT influences the clinical behavior of GIST. Most nonresponsive patients and almost all patients with early relapses were shown to carry an exon 9 KIT, PDGFR mutations or a wild type. Although KIT exon 11 missense mutations are found predominantly in lower-grade, favorable-outcome, GIST mutations involving deletion or duplication of multiple aminoacids in exon 11 have been related to poorer outcome [34]. Imatinib resistance has been related to survival pathways that circumvent KIT blockade linked to secondary mutations in KIT exons 13, 14, 17, or 18 which typically develop in tumors with primary exon 11 mutations leading to late progression in 50 to 70% of the patients [35].

Sunitinib, a multifunctional TK inhibitor, produces responses in patients with relapsing or resistant to imatinib [29]. *In vitro* inhibition of PI3K pathway in imatinib resistant cells arrests cell growth and induces apoptosis [35], suggesting that this may be a mechanism of action of sunitinib in imatinib-resistant patients. These observations are important as they relate to the fact that even specific targeted molecular therapies are not infallible or permanent and extensive further work lies ahead.

## 2. Molecular Biology of Sarcomas

The molecular findings in KS and GIST paved the way to an intensive search for molecular targets with a clinical translational potential. Bone and soft-tissue sarcomas encompass a very heterogeneous group of tumors with diverse pathological and clinical overlapping characteristics. Histomorphology distinguishes pleomorphic and nonpleomorphic tumors and spindle cell, epitheloid and small blue round cell tumors. With immunohistochemistry some tumors can be identified with epithelial or mesothelial line of differentiation. The advent of molecular testing has further assisted in the identification of more specific entities among this vast array of malignancies.

At a molecular level, a group of sarcomas are distinguished by specific molecular aberrations including somatic mutations, intergene deletions, gene amplifications, and reciprocal translocations. Others carry complex karyotypes and nonspecific genetic alterations. The majority of high-grade sarcomas with complex karyotypes have a high frequency of protein 53 (p53) and retinoblastoma protein (pRb) mutations as well as impairments in DNA repair and severe chromosomal instability, but no specific genetic alterations [36–40].

These molecular aberrations have undoubtedly improved the diagnostic capabilities to confirm the nature of many STS. However, the predictive and prognostic significance of these anomalies is hampered by the lack of standardization of methodologies and the small sampling amongst many of the several subtypes of sarcomas. Early reports that associate molecular abnormalities in ES and SS with prediction of outcome have been disputed in larger studies [41, 42].

### 2.1. Somatic Mutations

The gain-of-function KIT and PDGFR gene mutations in GIST are the most notable examples of this type of genetic abnormality in soft-tissue sarcomas.

### 2.2. Intergene Deletions

Rhabdoid tumors, which are highly aggressive and carry a poor prognosis, show partial or complete loss of the hSNF5/INI1 gene (chromosome 22q11.2). This gene is a core member of the SWI/SNF chromatin remodeling complex, and its loss leads to cell cycle progression. This abnormality is also present in 50% of malignant peripheral nerve sheath tumors and epithelioid sarcomas [37].

### 2.3. Amplifications

Amplifications of genomic regions are not specific for a given sarcoma subtype, but amplification of the murine double minute gene (MDM2) and the cyclin-dependent kinase 4 (CDK4) at chromosome 12q13–15 are highly characteristic in dedifferentiated liposarcomas (LPS) [40].

### 2.4. Reciprocal Translocations

Numerous translocations have been described in the past two decades that define 15–20% of sarcomas (Table 3). The first translocation identified in patients with Ewing sarcoma (ES) between the EWSR1 gene on chromosome 22q12 and FLI1 (Friend leukemia virus integration 1), a member of the ETS gene family of transcription factors on chromosome 11q24, was reported in 1992 [41]. This remains the most frequent abnormality, present in 85% of the patients. Other less frequent translocations have been described with other members of the ETS gene family. These translocations defined the ES family of tumors including the peripheral primitive neuroectodermal tumors (PNET) and became a critical diagnostic tool and a window to the pathogenesis of these and other sarcomas. The translocated genes give origin to a chimeric fusion gene encoding an aberrant transcription factor which alters several cell signaling pathways affecting proliferation and apoptosis leading to invasiveness and metastasis [43].

Several fusion proteins have been described resulting from translocations that involve the EWS family of genes and the ETS family in various types of sarcomas. ES includes fusion genes between EWS and FLI1 (85%) and ERG (10%) and rare cases involving ETV1, ETV4, FEV, and other ETS genes. Other gene products with a high homology to EWS can replace EWS in different fusions including CHN (or NOR1) in extraskeletal myxoid chondrosarcomas and CHOP (or CEBPE) in a proportion of myxoid LPS [38–40].

A second group of fusion genes are formed by non-EWS translocations and have been described in various types of sarcomas notably synovial sarcoma (SS), alveolar, rhabdomyosarcoma (ARMS), and myxoid LPS (Table 3).

These translocations have a tight specificity for a particular cell type. Gene fusions seem to only occur in susceptible cells at a given stage of development. Those cells surviving the abnormal translocation will eventually suffer malignant transformation. Accordingly, these fusion genes and their transcriptional targets may define the tissue lineage where the tumor originates, the phenotype of the transformed cells, and perhaps the clinical course of each subtype of sarcoma. ES is a striking example of lineage specificity [44].

The fusion genes are believed to promote carcinogenesis via stimulation or suppression of other genes involved in cell proliferation (i.e., upregulation of PDGF-C, CCDN1, and c-MYC), evasion of growth inhibition (via downregulation of CDK inhibitors and TGF-*β* receptor), escape from senescence by upregulation of hTERT, escape from apoptosis by repression of IGFBP-3 promoter, induction of angiogenesis by increasing VEGF and promoting invasion and metastases via matrix metalloproteinases (MMPs). Some fusion genes perform as autocrine stimulation loops. In dermatofibrosarcoma protuberans (DFSP), the growth factor platelet-derived growth factor B (PDGFB) is constitutively activated by the collagen, type I, alpha 1 (COL1A1) promoter in chromosome 17. In congenital fibrosarcoma the ETV6-NTRK3 fusion product (t(12; 15)(p13; q25)) results in constitutive activation of Ras/MAPK mitogenic pathway and PI3K/Akt pathway-mediated cell survival via the insulin-like growth factor 1 receptor [45, 46].

Overlapping of these groups is exemplified by clear cell sarcoma (CCS) and alveolar soft part sarcoma (ASPS) which are biologically linked by a common mechanism that upregulates expression of c-Met and tumorigenesis. These seemingly unrelated tumors dysregulate the MiT family of transcription factors and are referred as “MiT tumors” because of the involvement of the microphthalmia-associated transcription factor (MIFT) in their pathogenesis. In CSS the t(12; 22)(q13; q12) translocation fuses the EWS-ATF1 and constitutively activates AFT turning on several genes including MIFT [47]. ASPS is characterized by a non-EWS translocation between the ASPL locus on chromosome 17 and the TFE3 locus on the X chromosome (der(17)t(X; 17)(p11q25)) [48]. MITF, TFE3, TFEB, and TFEC comprise a family of transcription factors that share a highly homologous DNA binding and dimerization domain. These proteins bind identical DNA elements, suggesting that they may activate common downstream targets [49].

### 2.5. The Insulin-Like Growth Factor System (IGF)

The role of this pathway has been recognized as one of the major signaling pathways in the tumorigenesis of several sarcomas. The IGF consists of 3 main ligands: IGF-I, IGF-II, and insulin which bind to four types of membrane receptors, namely, IGF-1R, igf-2R, the insulin receptor (IR), and hybrid receptors. IGF-1R and Insulin receptors are 84% homologous and interact with each other to form heterodimers with high affinity to their ligands. In addition, there are IGF circulating binding proteins (IGFBP) that modulate the availability of the free ligands to the receptors. The concentration of the ligands and the binding proteins are under the influence of growth hormone and liver synthesis. The binding of ligand and receptor activates intracellular signaling cascades via the PI3K and MAPK pathways. Abnormalities among or along these numerous molecules have been described in different human malignancies and particularly in sarcomas [50, 51].

Several sarcoma fusion genes and other molecular anomalies have been shown to upregulate ligand (IGF-1 or IGF-2) or receptor (IGF-1R) expression and, in ES, to downregulation of IGFBP-3 (Table 4) The IGF system, particularly the IGF-1R, has been validated both *in vitro* and in experimental animal models as a central participant in the induction of malignant transformation in ES, RMS, leiomyosarcoma (LMS), and other sarcomas. Furthermore, preliminary clinical observations have related markers of the IGF system to tumor behavior and prognosis [51].

### 2.6. The PI3K Pathway

**Figure 1:**
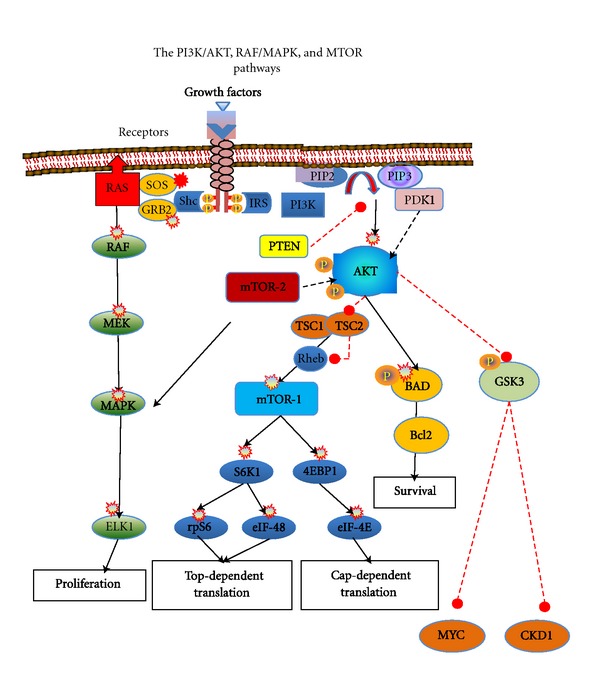
Several growth factor signals activate cell membrane receptor tyrosine kinases leading to activation of downstream interacting signal transduction pathways (PI3K/AKT, RAF/MAPK, and MTOR). Within the RAF/MAPK pathway, activated receptors lead to SHC-mediated activation of RAS and propagation of signalling through RAF, MEK (a.k.a. MAP2K), and MAPK (a.k.a. ERK). Activated MAPK forward signals to the nucleus that regulate proliferation, differentiation, angiogenesis, and cell survival. A second essential signalling pathway is the PI3K/AKT pathway. PI3K catalyzes triple phosphorylation of phosphatidylinositol (PI) AKT in conjunction with its PDK1. AKT is a key molecular “node” acting as a master switch, which triggers multiple downstream signaling pathways including mTOR pathway activation. A key regulatory enzyme is PTEN that modulates PI3K and SHC phosphorylation. Activated AKT signals a number of mitogenic processes promoting proliferation and increased cell survival, antiapoptotic signals, and upregulation of cell-cycle proteins (cyclin D1 and CDK4).

Heightened expression of IGF-1R has been related to loss of transcriptional repressors (p53) or increase in transcriptional activators in various human cancers. Upregulation of IGF-1R results in sustained activation of downstream PI3K and MAPK pathways (Figure 1). The carcinogenic potential of abnormal or unregulated PI3K/AKT or RAS/MAPK pathways' signaling is related to their regulatory role in cell cycle progression, cell growth and proliferation, differentiation, and apoptosis [50, 51]. Activation of PI3K turns on AKT, key molecular “node” acting as a master switch, which triggers multiple downstream signaling pathways (including mTOR activation) promoting proliferation and increased cell survival, antiapoptotic signals and upregulation of cell-cycle proteins (cyclin D1 and CDK4). Recent experimental evidence supports the central role of AKT in STS with complex karyotypes. In a mouse model using and an adenovirus with Cre-recombinase and conditional mutations of Kras and p53 over 90% of the animals developed high grade sarcomas, resembling malignant fibrous histiocytoma (MFH) [52]. Another model using mice genetically inactivated for pTEN (phosphatase and tensin homolog) over 80% of the animals developed abdominal LMS [53].

### 2.7. MAPK

Overexpression and activation of the MAPK pathway (MKK) has been demonstrated in several epithelial malignancies and related to tumor proliferation and metastases. This pathway is coactivated by IRS system with PI3K but its role in the genesis of sarcoma is less defined. Growth factor-induced activation of Ras leads to the activation of Raf, followed by the activation of MAPK/ERK kinases (MEK1-MEK2) and lastly the activation of ERK1 and ERK2 (Figure 1). The latter proteins regulate cell proliferation, survival, differentiation, and migration. In one study, RAF1 and MEK 1/2 mRNA were detected in STS cell lines and OS specimens and demonstrated dose- and time-dependent inhibition of cell growth when treated with a MEK inhibitor [54]. Similar observations have been reported in SS where TK inhibition with sorafenib inhibited the MKK pathway, downregulated cyclin D1 and pRb levels, caused G1 arrest, and induced apoptosis [55]. In a xenograft model, MKK signaling was necessary for tumor growth and vascularization and treatment with anthrax lethal toxin (LeTx) produced extensive tumor death and antiangiogenic effects. LeTx contains lethal factor (LF), a zinc metalloprotease that cleaves and inactivates several MKK proteins, suggesting that MKK had a predominantly proangiogenic effect in this model [56].

### 2.8. The Role of mTOR Pathway

Activation of PI3K-AKT is a convergence point of activation of growth factor receptors driving the growth of various sarcomas (Figure 1). Intrinsic activation of the mTOR pathway in sarcomas is the result of abnormal signaling of these pathways [57]. Other mechanisms explaining over activation of mTOR involve the loss of regulatory inhibitory gene activity of the tuberous sclerosis complex (TSC) proteins or PTEN inactivation by methylation of the promoter, gene mutation, or allelic deletions such as those reported in perivascular epithelioid cell tumors (PEComas). Loss of LKB1 protein also leads to hyper activation of mTOR signaling in a manner similar to the loss of PTEN [58–60].

### 2.9. Angiogenesis

Angiogenesis is regulated by equilibrium between proangiogenic (i.e., VEGF, FGF, EGF, PDGF, HIF) and antiangiogenic factors (i.e., angiostatin, IFN, thrombospondin, and interleukins 1, 4, 12, 18, and 21) which are produced by both the malignant cells and the microenvironment including endothelial cells, fibroblasts, and immune cells. Vascular endothelial growth factor pathway plays a dominant role in the pathogenesis and biology of STS. VEGF is overexpressed in 25% of tumors, and high VEGF is associated with an increased risk of metastases and poorer prognosis. Strong VEGF expression is often present in tumors rich in vasculature and epithelioid features such as epithelioid sarcoma, KS, and ASPS. Serum VEGF levels have been strongly correlated with tumor grade and mass and usually poorly differentiated tumors. STS with high VEGF expression are associated with resistance to chemotherapy [61, 62]. As with IGF, the VEGF downstream pathway involves PI3K and MAPK indicative of intermingle of cell membrane signals. Distinct from these pathways is the VEGF-induced activation of PGLG1 which exerts proangiogenic effects via protein kinase C (PKC) [61].

Other proangiogenic factors are upregulated in STS including PDGFR, MMP-2, and Notch-1 and Notch-4, basic FGF and angiopoietin-2 with certain variability according to type and grade of the sarcoma. For instance, fibrosarcoma and LMS showed the highest bFGF levels [63]. Some chromosomal translocations and their fusion proteins can act as transcription factors for promoters of the VEGF gene as is the case of the highly vascular ASPS. Similarly the activation of mTOR may promote angiogenesis via control of the hypoxia inducible factor (HIF)-1*α* and mTOR inhibition results in an antiangiogenic effect [58].

### 2.10. Telomeres

Cell senescence has been considered a hallmark of cancer and has been related to chromosomal telomere maintenance mechanisms currently under scrutiny in several malignancies. In a LPS model, using high-resolution DNA mapping array, high level of genome instability and genetic amplifications were identified. In contrast to most stem cells and other cancer cells, that use reverse transcriptase telomerase for telomere maintenance, sarcoma cells activate the alternative lengthening of telomeres (ALT) mechanism as often as telomerase. ALT positive LPS have higher genetic instability and a worse prognosis than non-ALT tumors [64]. A genetic change, the deletion of chromosome 1q32.2-q44, is seemingly specific to the activation of ALT mechanism in this model [65].

### 2.11. Hedgehog (Hh) Pathway

Activation of Hh pathway results in stimulation of a wide range of prosurvival transcription factor genes. Abnormal activation of Hh pathway has been implicated in the genesis of various cancers particularly basal cell carcinoma, medulloblastomas, and RMS. Activation of Hh is manifested by the expression of several proteins (Ptch1, Gli1, Gli3, and Myf5). These markers are expressed by embryonal RMS and fusion gene-negative alveolar RMS, whereas FOXO1-PAX3/PAX7 positive ARMS are not [64]. Preliminary observations have shown augmented Hh signaling in cancer stem cells as well as in stromal nonmalignant cells surrounding malignant tumors and may constitute another cofactor in the genesis of sarcomas and other cancers [66].

### 2.12. Cancer Stem Cells and Mesenchymal Cells in Sarcoma

A growing body of evidence suggests the existence of cancer stem cells (CSCs), pluripotential stem cells that can perpetuate the generation or renewal of tumor forming cells in solid tumors [67]. The first evidence of cancer stem cells in sarcoma was reported in 2009. Using surgically resected ES primary tumors, a population of CD133 positive cells fulfilling *in vitro* and *in vivo* criteria of CSC was identified. These criteria included the capacity to generate and sustain tumor growth in a xenograft model and to differentiate, *in vitro,* along adipogenic, osteogenic, and chondrogenic lineages [68]. These data are supportive of the mesenchymal stem cell (MSC) origin of ES. Thus, ES-initiating cells seem to conserve the properties of their putative cell precursors. In this model, the expression of the EWS-FLI-1 fusion protein in MSC cells was sufficient to develop ES-like tumors. Lastly, the CD133+ CSC studied showed significantly higher expression of OCT4 (octamer-binding transcription factor 4) and NANOG genes known to be critically involved in self-renewal of embryonic stem cells [68].

While testing other sarcoma-inducing fusion genes, only FUS-CHOP-expressing transfected cells were found to generate tumors resembling human myxoid LPS. Together with the findings in ES, these data raise the intriguing possibility that ES and MLS may originate from closely related mesenchymal cells, but at different anatomical locations. Perhaps the microenvironment (bone in ES, soft tissue in MLS) contributes to the line of differentiation that the MSC follow according to the adaptability of the transformed cell to survive in a given tissue environment [69].

### 2.13. Mesenchymal-Epithelial Transition in Sarcoma

Another puzzling and intriguing feature of certain sarcomas is the apparent spontaneous transition from a mesenchymal tumor to an epithelial enriched tumor (mesenchymal-epithelial transition (MET)). There is growing evidence supporting the role of epithelial-to-mesenchymal transition (EMT) during carcinoma progression and metastases linked to the consequences in cell morphology, cell-to-cell adhesion, cell motility, and plasticity to migrate and growth in the extracellular matrix. The mesenchymal to epithelial transition (MET) in sarcoma progression is considerably less well studied. Triggering of MET has been shown to be induced by c-met proto-oncogene, a TK receptor for HGF/SF. Increased expression of this protein leads to epithelial differentiation. Epigenetic regulation of DNA methylation has also been related to the MET [70].

This phenomenon is particularly notable in SS. Monophasic SS is entirely composed of spindle cells with or without solid epithelial areas, whereas the biphasic SS contains a lining of epithelial cells amongst the spindle cells [71]. At a molecular level, the SYT-SSX1 fusion occurs five times more frequently in biphasic SS exhibiting MET in comparison to SYT-SSX2 in monophasic SS. These data indicates a possible role of SYS-SSX1 and SYT-SSX2 in the MET phenomenon by interacting with transcription receptors (snail or slug, resp.) interfering with the E-cadherin gene and triggering the MET epithelial differentiation program in the affected SS cells [72].

Similar observations have been described in a chondrosarcoma model, where upregulation of 4 distinct epithelial markers and the downregulation of snail were found. Loss of DNA methylation was demonstrated in maspin and 14-3-3*σ* genes leading to increased expression of these 2 epithelial-specific genes during chondrosarcoma genesis. These results support the relationship between MET and an epigenetic switch in chondrosarcoma [73].

### 2.14. MicroRNA in Sarcomas

The discovery of microRNA (miRNA) has been one further step into the molecular universe of gene regulation and cell biology and consequently into the biology of cancer cells and oncogenesis. There is an intense search for miRNA profiles in diverse human cancers to better understand the role of these minute RNA molecules in cancer control and to explore potential therapeutic implications [74].

Preliminary work in sarcomas has disclosed unique miRNA expression signatures according to the histological types seemingly reflecting the cell lineage and differentiation status of the tumors [75]. Hierarchal clustering of 87 miRNAs disclosed four main groups, whereby almost all SS, RMS, LMS, and GIST were grouped distinctively. In GIST, miR-221 and miR-222 had low expression, suggesting that the decreased suppressive activity of these miRNAs allows increased translation of KIT. The miRNAs that play a major role in myogenesis, miR-1, miR-133A, and miR-133B, were overrepresented in LMS, whereas miR-335, involved in skeletal muscle differentiation, was present in ARMS. In SS, miR-143 whose target is ERK5 (MAPK7), was expressed at very low levels suggesting that this miRNA may be involved in the expression of the SYT-SSX1 oncoprotein [76]. Others have linked the expression of miR-200 to mesenchymal-epithelial differentiation often reported in SS [77].

In a recent study exploring the genesis of CSC phenotype in ES, Riggi and collaborators [78] published evidence that repression of miRNA-145 and expression of the EWS-FLI-1 fusion gene were both necessary to induce transformation. They showed evidence that their common target may be the SOX2 gene. This gene is known to code for transcription factors required for the development of pluripotent stem cells. These observations provided valuable insight into the mechanisms, whereby a single oncogene (EWS-FLI1) can reprogram cells towards CSC.

## 3. Therapeutic Implications

**Figure 2:**
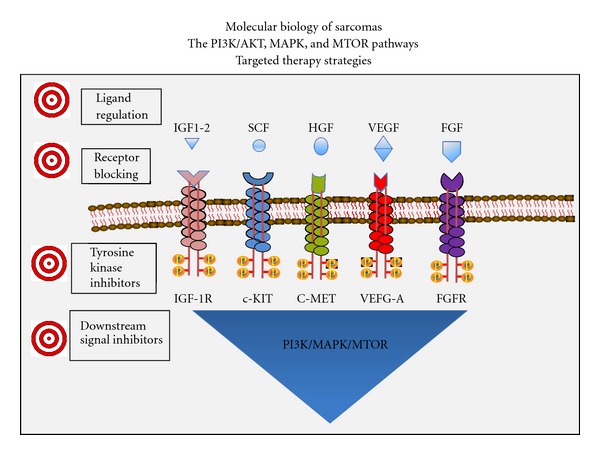
Several ligands (IGF1/2, SCF, HGF, VEGF, and FGF) activate cell membrane receptor tyrosine kinases (IGF1R, C-KIT, C-MET, VEGFR-A, and FGFR) triggering shared interacting signal transduction pathways (PI3K/AKT, RAF/MAPK, and MTOR). The availability of ligands via paracrine secretion (IGF1), autocrine loops (VEGF), or ligand binding (IGFBPs) can modulate activation of these pathways. Molecular anomalies of the receptors or the downstream signals lead to constitutive activation or dysregulation of these signals. Multiple cell processes including proliferation, differentiation, angiogenesis, and survival are promoted as a result of the activation of these main pathways in sarcomas and other neoplasms. Actionable targets are listed that may interfere with the abnormal signalling and could result in beneficial biologic and clinical effects.

Targeting the multiple molecular pathways and mechanisms summarized above is one of the areas of intense basic and clinical research. The objective is to find means to modify tumor behavior and to find long awaited clinical therapeutic options for these patients. Search for antagonistic antibodies, TK inhibitors, and inhibitors of downstream molecules of the PI3K, MAPK, and mTOR paths are at the forefront of these efforts (Figure 2). The preliminary clinical trials have not yet crystallized into therapeutic breakthroughs despite solid preclinical evidence, suggesting a broad range of favorable biological effects from the inhibition of these pathways.

### 3.1. IGF-1R Antibodies

Preclinical data proves that effective blockade of IGF-1 and IGFII ligands to the IGF-1R is feasible [79]. Monoclonal antibodies against the IGF-1R have been the favored approach to date. Phase I and II studies of IGF1R antagonists figitumumab, cixutumumab, AMG479, R1507, and SCH 717454 either alone or in combination with other agents, are currently under clinical investigation for patients with sarcomas.  Table 5 summarizes the results of reported phase I/II studies with anti-IGF-R1 agent [79–83].

Further, IGF-1R has been implicated in chemotherapy resistance from *in vitro* work with malignant cells. In an ES tumor model, combination of *ADW742* with imatinib, vincristine, and doxorubicin induced a significant reduction of tumor cell growth, mainly by increasing apoptosis [84]. Similarly, *NVP-AEW541* led to cytotoxicity and induced apoptosis in imatinib resistant or wildtype GIST [85]. Finally, there is experimental evidence that bidirectional crosstalk between the IGF system and the erbB family of receptors may confer means of escape or resistance to target therapy of these receptor pathways [51]. Thus, the use of combined therapies aiming to block several pathways simultaneously is being actively investigated in various tumors.

### 3.2. TKs Inhibitors and Antiangiogenesis

As described above, TKs account for a large number of defective signaling pathways in sarcomas. Based on the success of imatinib in GIST, TKs inhibitors are of major interest in other sarcomas. Sunitinib has been shown to be effective in imatinib-resistant GISTs [86]. Patients with chordomas, desmoids, and DFSP tumors reportedly responded to imatinib in small trials and isolated case reports, likely via inhibition of PDGFR [87].

 Initial attempts intended to block signaling pathways with other available agents known to interfere with either cellular receptors or TKs have not met with evidence of major or consistent clinical benefit. Phase II studies with EGFR inhibitors (gefitinib or erlotinib) showed no clinical activity in SS or malignant nerve-sheath tumors. Use of trastuzumab in ES and OS, alone or in combination with chemotherapy, has had no therapeutic benefit. Phase II studies with sunitinib in non-GIST sarcomas have only shown occasional objective responses, but disease stabilization over 12 weeks was noted in several histologies [87]. Sorafenib, but not sunitinib, was reported to produced a 14% PR rate and a median OS of 14.3 months in angiosarcomas [88]. Some sarcoma subtypes with a predominant vascular connective tissue component such as angiosarcomas, intimal sarcomas, and hemangiopericytomas may arise from endothelial cells, suggesting that proangiogenic proteins may be relevant to their growth and potential treatment targets. Several multitargeted receptor TK inhibitors with antiangiogenic effects are currently on clinical trials.  Table 6 shows published or reported data on various new multitargeted TKs [89–93].

*ABT-510*, a peptide that mimics the antiangiogenic activity of thrombospondin-1, was tested in a phase II trial in patients with advanced STS. Approximately 50% of patients achieved SD, with 1 objective response. *Perifosine* an AKT inhibitor has been tested in phase I and phase II trials in patients with advanced STS with inconsistent results. No objective responses were observed but 27% of patients experienced SD in one study with a 5% PR rate and 45% of patients experienced SD for >4 months in a retrospective evaluation [94].

### 3.3. mTOR Inhibitors

The clinical impact of analogs of rapamycin (rapalogs) in the management of renal cell carcinoma has confirmed the antitumor potential of mTOR pathway interference [95]. The participation of mTOR in the genesis of sarcoma is related to the primordial role of the IGF system in these tumors. Thus, mTOR inhibitors were a natural choice to test clinically in sarcomas.

Rapamycin exert its biological effects by forming a complex with FKBP12 which binds to the FK-rapamycin binding domain of mTOR inhibiting the function of mTORC1-mediated signal pathway and resulting in a direct antiangiogenic effect [58]. A number of preclinical studies with various rapalogs paved the way to subsequent human trials. These have included *in vitro* and *in vivo* observations in mouse xenograft models using sirolimus, temsirolimus, everolimus, and ridaforolimus. Of relevance, temsirolimus inhibits HIF-1*α* translation and interferes with VEGF protein expression in RMS demonstrating suppressed tumor growth via anti-angiogenesis [96]. Other models have suggested that sarcomas associated with PTEN loss or inactivation may be particularly susceptible to the therapeutic effects of mTOR inhibitors [58, 59]. Rapalogs have been shown to be less effective or ineffective in the presence of KRas mutations or overexpression of Bcl2, whereas tumors with cyclin D1 expression and “angiogenesis addiction” are more susceptible [97]. 

Phase I/II clinical trials with several rapalogs and combinations aim to determine efficacy in sarcoma patients. Ongoing studies include testing efficacy of sirolimus in KS, temsirolimus, and valproic acid in OS and STS; or combinations with vinorelbine for uterine sarcoma, liposomal doxorubicin or irinotecan for recurrent or refractory sarcomas [96, 97].  Table 7 summarizes data on early phase studies with rapalogs. Ridaforolimus is a nonprodrug rapalog that has shown promising clinical activity in STS [98, 99]. Despite disputed clinical impact (the difference in median PFS was only 3 weeks), this drug is awaiting approval in 23 countries including the FDA in the US based on these studies.

### 3.4. Combinatorial Studies

A number of early clinical studies explored combination of agents intended to block redundant or cross-talking molecular pathways or to circumvent chemotherapy resistance. These include everolimus with imatinib, phase II study in imatinib-resistant GIST; everolimus and figitumumab; temsirolimus with cixutumumab in ES (65% of patients had tumor reduction >20%) ridaforolimus and doxorubicin; everolimus and pegylated liposomal doxorubicin, mTOR inhibitors and hormonotherapy, and many others will follow [96–99]. There is also a growing interest in the use of other agents including melatonin, metformin, celecoxib, statins, and others which have been shown to modulate molecular signals in the pathways involved in tumor growth. Melatonin has been shown to have a broad range of activities resulting in an oncostatic effect *in vitro* and *in vivo *including suppression of tumorigenesis in methylcholanthracene-induced fibrosarcomas in mice [100]. In an ES *in vitro* model, melatonin has been shown to induce apoptosis and synergism when combined with chemotherapeutic agents [101]. Metformin reduces insulin level by decreasing insulin resistance a favorable effect on cancer cells dependent on the IGF system. A direct inhibitory effect of metformin on cancer cell growth has also been reported and has been associated to a regulatory role in the AMP-activated protein kinase (AMPK) and mTOR pathways [102].

## 4. Conclusions

A wealth of information is accumulating at a rapid pace that, undoubtedly, will continue to contribute to the further understanding of the molecular biology of sarcomas. There are, however, enormous challenges ahead, particularly in the clinical translation of these discoveries. Despite the success noted in KS and the success of imatinib treatment in GIST, only modest gains have been attained in our attempts to alter or modulate growth of tumor in most other sarcomas. It is clear that the rarity of this vast number of pathological entities complicates the clinical application of the newly developing agents. But the existence of fairly specific genetic abnormalities, mainly specific translocations and a generation of discernible fusion genes in various sarcomas make research in these tumors an attractive mold for advancing studies of solid tumors.

Finding key molecular “nodes” or pathways that are common amongst these tumors will help to further understand the pathogenesis of sarcomas and may help improve current therapeutic choices. Yet, the redundancy of these molecular cascades, the loopholes and the fact that tumors may find adaptive escape routes necessitate incessant search for deeper understanding of the signaling pathways in the context of all the hallmarks of cancer discussed above. The concept of system biology and the development of computational and mathematical models are areas that will facilitate the organization of thoughts directing future research strategies.

Clinically, biologic effects are largely cytostatic and transient which indicate the need to devise new strategies to reach long-term clinical control and potential cure of some of these entities. Newer clinical trials are beginning to modify the chemotherapy-oriented criteria of response and criteria in the designing of clinical studies [103]. Emphasis is being placed in the selection of patient populations to avoid the pitfalls of unselected trials that have often missed the target in the past. There is a clear trend to consider progression-free survival (PFS) as a more relevant endpoint when assessing the antitumor effects of molecular targeted therapies. It has been suggested that first-line treatments should achieve PFS rates of >30% at 6 months for the results to be considered clinically meaningful in phase II trials in STS [86].

Assessment of “metabolic” response by FDG-PET is being examined as a better mean of assessing antitumor activity of targeted therapies as opposed to traditional RECIST-defined responses. Support for this is coming in many fields including sarcoma trials [104]. FDG-PET has been shown superior to CT in predicting early response and monitoring of response and progression to imatinib therapy in GIST and other STS [105, 106]. Furthermore, FDG-PET may also have applicability as an early pharmacodynamic marker of molecular targeted therapies [106].

Although the concept of “personalized” therapy has been overestimated there is clearly a need to continue searching for uniqueness among subgroups of patients, particularly in sarcomas, this most heterogeneous collection of diseases. Cardinal to these efforts is the search for molecular markers that identify patients most likely to benefit from a given intervention as well as to monitor the biological effects of the treatment or to identify the optimal dose of these agents. It is necessary to dismiss the chemotherapy-driven concept of maximum tolerated dose and find instead the optimal biologic dose of the agents being tested. So far the identification of clinically relevant molecular markers has been daunting but progress in the area is expected as methodologies to examine efficiently multitude putative “markers of interest” are developed.

These interventions are not strictly tumor-specific and elicit cellular and clinical toxicity and may hamper our enthusiasm to some extent as we have already witnessed in other fields including RCC, melanoma, colon, breast, and other cancers. The identification of more precise targets and refinement of the specificity of targeted agents is required to make progress in this field. These targets should be those that are essential for the malignant cells to subsist, a concept that has been termed “oncogene addiction.” But even well-characterized mutations can have different molecular isoforms that could alter the specificity and durability of the binding to the therapeutic agent or render the agent less efficacious. Identification of precise “pockets” within the abnormal targeted molecule may also improve the therapeutic index of future targeted therapies.

Enormous clinical challenges lie ahead, but a new world of possibilities is opening. Combination strategies are very attractive because of the multitude of potential targets and because of the different properties and mechanisms of action of drugs in our growing arsenal. However, the combination of some of these agents has already shown additive toxicities without an additional antitumor effect. Antagonism has been observed in some combinations that seemed logical and promising and, although observed in preclinical models, we are yet to prove synergism when combining these agents. Combination therapies should not be limited to molecular-targeted drugs as we can judiciously combine these with chemotherapeutic agents. Furthermore, these combination strategies must include efforts to modulate the microenvironment of the tumor and the immune system to attain a full and comprehensive approach to the control of the malignant growth in sarcomas and other cancers.

## Figures and Tables

**Table 1:** HHV-8 Genes and role in Kaposi Sarcoma.

Latent genes	Effects	Other properties
V-cyclin (ORF 72)	Constitutively activates CDK6 Interferes with cyclin-dependent cell cycle arrest Bypasses inhibitory controllers of cell cycle regulation (p16, p21, and p27)	Phosphorylates pRb; Releases E2F Activates S-phase genes
v-FLIP (ORF 71)	Competes with proapoptotic signals mediated via FAS-FADD	Oncogenic
LANA (ORF 73)	Blocks tumor suppressor genes p53 Competes with E2F transcription factor for binding to pRb	Controls genes triggering the switch to lytic phase Oncogenic
Kaposin (K12)	Upregulates PORX1; induces reprogramming of vascular endothelial cells	Oncogenic
K10.1 (LANA-2)	Blocks IFN- and IRF-mediated transcriptional activation; binds p53?	

Lytic genes	Effects	Other properties

vIL-6 (ORF K2)	Activates gp130 independently of IL-6R Activates NF*κ*B; constitutively activates GPCR. Activates JAK1 and STAT 1/3 pathways Induces transcription of VEGF and MMP-9	Autocrine and paracrine loops Oncogenic
vGPCR (ORF 74)	Constitutively activate GPCRs; binds IL-8	Oncogenic Angiogenic
vMIP-I and vMIP-II (ORF K4)	CCR-3 and 4 agonist Broad spectrum cytokine receptor antagonist.	Entry coreceptor for HIV-1 Angiogenic
vBcl2 (ORF 16)	Antiapoptotic activity Inhibits bax	Oncogenic

**Table 2:** HIV and Kaposi Sarcoma. HHV-8 genes activated by HIV-Tat.

HHV-8 genes	Effects	Other properties
vGPCR (ORF 74)	Constitutively activates GPCR Promotes synthesis of VEGF	Oncogenic Angiogenic
vBcI2 (ORF 16)	Antiapoptotic activity Inhibits bax	Oncogenic
vIRF-1 (ORF K9)	Interferon regulatory factor homolog Activates the c-myc oncogene	Antagonizes IFN-mediated antiviral immunity

**Table 3:** Reciprocal translocations in sarcomas.

Tumor	EWS translocations	Fused genes	Incidence (%)
Ewing sarcoma/PNET	t(11; 22)(q24; q12) t(21; 22)(q22; q12) t(7; 22)(p24; q12) t(17; 22)(q12; q12) t(2; 22)(q33; q12)	EWS-Fli1 EWS-ERG EWS-ETV1 EWS-E1AF EWS-FEV	85 10 Rare Rare Rare
Desmoplastic small round cell tumors	t(11; 22)(q13; q12)	EWS-WT1	75
Myxoid liposarcoma	t(12; 22)(q13; q12)	EWS-CHOP	5
Clear cell sarcoma	t(12; 22)(q13; q12)	EWS-ATF1	>90
Myxoid liposarcoma	t(12; 16)(q13; q11)	FUS-CHOP	95
Extraskeletal myxoid chondrosarcoma	t(9; 22)(q22; q11) t(9; 17)(q22; q11)	EWS-CHN RBP56-CHN	75 20

Tumor	Non-EWS Translocations	Fused genes	Incidence (%)

Synovial sarcoma	t(X; 22)(p11.23; q11) t(X; 18)(p11.21; q11)	SYT-SSX1 SYT-SSX2	65 35
Alveolar Rhabdomyosarcoma	t(2; 23)(q35; q14) t(1; 13)(q36; q14)	PAX3-FKHR PAX7-FKHR	75 10
Congenital fibrosarcoma	t(12; 15)(q13; q25)	ETV6-NRTK3	Unknown
Alveolar sort part sarcoma	t(X; 17)(p11; q25)	ASPSCR1-TFE3	99

**Table 4:** The IGF System in sarcoma pathogenesis.

Tumor	Abnormal genes	Involved ligand	Involved receptor	Other properties
Ewing sarcoma/PNET	EWS-FLI1	IGF-1	IGF-1R	Downregulates IGFBP-3
Synovial sarcoma	SYT-SSX1/SYT-SSX2	IGF-2	IGF-1R	
Alveolar rhabdomyosarcoma	PAX3-FKHR/PAX7-FKHR	IGF-2	IGF-1R	Autocrine loop
Desmoplastic small round cell tumors	EWS-WT1	?	IGF-1R	
Congenital fibrosarcoma	ETV6-NTRK3	IGF-2	IGF-1R	
Embryonal rhabdomyosarcoma	LOH 11p15.5 pften	IGF-2	IGF-1R	Autocrine loop
Leiomyosarcoma	Complex karyotypes	IGF-2	IGF-1R	PI3K highly activated
Osteosarcoma	Complex karyotypes	IGF-1, IGF-2	IGF-1R	
Kaposi sarcoma	Complex karyotypes	?	IGF-1R, IR-A	
GIST	Lacking KIT, PDHFR mutations	IGF-1, IGF-2	IGF-1R	Poorer Prognosis

**Table 5:** Current Anti-IGF treatment of sarcomas.

Monoclonal antibodies	Human trials	Disease control rate (CR, PR, and SD)	Comments
Figitumumab CP-751, 871	ES/STS	10/28 (35%)	Anti-IGF-IR
Cixutumumab	STS	22/37 (59%)	Liposarcomas; block hybrid receptors
Robatumumab SCH 717454	Preclinical	OS, RMS	Anti-IGF-IR
Ganitumab AMG479	ES	2/15 (13%)	
RI 507	ES	18/125 (14.4%)	No SD included

TK inhibitors	Status	Target disease	

NVP-AEW541	Preclinical	ES/STS/GIST	Synergy with chemoRx
NVP-ADW 742	Preclinical	ES	Synergy with imatinib
BMS-536924	Preclinical	STS	ATP-competitive IGF-IR

Others	Status	Target disease	

Nordihydroguaiaretic acid (NDGA)	Preclinical	STS	Disrupts IGF-1R; blocks HER-2

**Table 6:** Current clinical trials with tyrosine kinase inhibitors in sarcomas.

Agent	Human trials	Clinical benefit rate (CR, PR, and SD)	Targets: notes
Imatinib	GIST	85% (5% CR; 45% PR)	KIT. FDA Approved
Sunitinib	GIST	65% (7% PR)	VEGFR-1, VEGFR-2, VEGFR-3, PDGFR a/*β*, KIT. FDA Approved
Sorafenib	Angiosarcoma, GIST	14%	VEGFR-2, VEGFR-3, PDGFR, c-RAS, b-RAF, KIT
Pazopanib	Palette STS	PFS = 20 versus 7 weeks (*P* = 0.0001)	VEGFR-1, VEGFR-2, VEGFR-3, PDGFR-a/*β*, KIT
Brivanib	STS ASCO 2011	30% overall 3 PR in angiosarcomas	FGF and VEGF (FGF-positive did better)
Cediranib	STS ASCO 2011	78% (43%, PR) Alveolar soft part sarcoma	VEGFR-1, VEGFR-2, and VEGFR-3
Tivantinib	STS ASCO 2011	80% (5%, PR) Clear cell sarcoma, ASPS	c-Met
Axitinib	STS, angiosarcoma	N/A	VEGFR-1, VEGFR-2, and VEGFR-3

**Table 7:** Current clinical trials with rapalogs in sarcomas.

Agent	Human trials	Clinical benefit rate (CR, PR, and SD)	Notes
Temsirolimus	GIST ASCO 2010	GIST = 4/15 (27%) STS = 2/15 (13%)	Refractory GIST
Temsirolimus	Phase II STS Mayo Clinic	5%	
Sirolimus	Kaposi sarcoma	15/15 (100%)	Posttransplant KS Dermal lesions
Ridaforolimus	Phase II-STS	61/212 (29%; 2% PR)	IV formulation
Ridaforolimus	SUCEED trial	PFS: HR = 0.69 (*P* < 0.0001) (22.4 versus 14.7 weeks)	Maintenance after ChemoRX
